# Expanding the phenotype and genotype spectrum of *TAOK1* neurodevelopmental disorder and delineating *TAOK2* neurodevelopmental disorder

**DOI:** 10.1016/j.gim.2024.101348

**Published:** 2024-12-27

**Authors:** Nour Elkhateeb, Renarta Crookes, Michael Spiller, Lisa Pavinato, Flavia Palermo, Alfredo Brusco, Michael Parker, Soo-Mi Park, Ariana Costa Mendes, Jorge M. Saraiva, Trine Bjørg Hammer, Lusine Nazaryan-Petersen, Tahsin Stefan Barakat, Martina Wilke, Elizabeth Bhoj, Rebecca C. Ahrens-Nicklas, Dong Li, Tomoki Nomakuchi, Eva H. Brilstra, David Hunt, Diana Johnson, Sahar Mansour, Kathryn Oprych, Sarju G. Mehta, Konrad Platzer, Franziska Schnabel, Henriette Kiep, Helene Faust, Gillian Prinzing, Kimberly Wiltrout, Jessica A. Radley, Alvaro H. Serrano Russi, Isis Atallah, Belinda Campos-Xavier, David J. Amor, Angela T. Morgan, Christina Fagerberg, Ulla A. Andersen, Charlotte B. Andersen, Emilia K. Bijlsma, Lynne M. Bird, Sureni V. Mullegama, Andrew Green, Bertrand Isidor, Benjamin Cogné, Janna Kenny, Sally A. Lynch, Shauna Quin, Karen Low, Theresia Herget, Fanny Kortüm, Rebecca J. Levy, Jennifer L. Morrison, Patricia G. Wheeler, TaraChandra Narumanch, Kristina Peron, Nicole Matthews, Jillian Uhlman, Lauren Bell, Lewis Pang, Ingrid Scurr, Rebecca S. Belles, Bonnie Anne Salbert, Gerald Bradley Schaefer, Sarah Green, Andrea Ros, Agustí Rodríguez-Palmero, Tanja Višnjar, Karin Writzl, Pradeep C. Vasudevan, Meena Balasubramanian

**Affiliations:** 1 Department of Clinical Genetics, Cambridge University Hospitals NHS Foundation Trust, Cambridge, United Kingdom; 2 Sheffield Diagnostic Genetics Service, Sheffield Children’s Hospital NHS Foundation Trust, Sheffield, United Kingdom; 3 Department of Medical Sciences, University of Turin, Turin, Italy; 4 Institute of Oncology Research (IOR), Bellinzona Institutes of Science (BIOS^+^), Bellinzona, Switzerland; 5 Faculty of Biomedical Sciences, Università della Svizzera Italiana, Lugano, Switzerland; 6 Department of Neurosciences Rita Levi-Montalcini, University of Turin, Turin, Italy; 7 Medical Genetics Unit, Città della Salute e della Scienza University Hospital, Turin, Italy; 8 Sheffield Clinical Genetics Service, Sheffield Children’s NHS Foundation Trust, Sheffield, United Kingdom; 9 Medical Genetics Department, Hospital Pediátrico de Coimbra, Unidade Local de Saúde de Coimbra, Coimbra, Portugal; 10 University Clinic of Pediatrics, Faculty of Medicine, University of Coimbra, Portugal; 11 Clinical Academic Center of Coimbra, Hospital Pediátrico de Coimbra, Unidade Local de Saúde de Coimbra, Coimbra, Portugal; 12 Department of Epilepsy Genetics and Personalized Medicine, Danish Epilepsy Centre, Dianalund, Denmark; 13 Department of Genetics, Rigshospitalet, Copenhagen, Denmark; 14 Department of Cellular and Molecular Medicine, Faculty of Health and Medical Sciences, University of Copenhagen, Copenhagen, Denmark; 15 Department of Clinical Genetics, Erasmus MC University Medical Center, Rotterdam, The Netherlands; 16 Discovery Unit, Department of Clinical Genetics, Erasmus MC University Medical Center, Rotterdam, The Netherlands; 17 ENCORE Expertise Center for Neurodevelopmental Disorders, Erasmus MC University Medical Center, Rotterdam, The Netherlands; 18 Division of Human Genetics, Children’s Hospital of Philadelphia, Philadelphia, Pennsylvania; 19 Department of Genetics and Brain Center, University Medical Center Utrecht, The Netherlands; 20 Wessex Clinical Genetics Service, Princess Anne Hospital, University Hospital Southampton NHS Trust, Southampton, United Kingdom; 21 South West Thames Regional Genetics Service, St George’s University Hospitals NHS Foundation Trust, London, United Kingdom; 22 School of Biological and Molecular Sciences, St George’s University of London, London, United Kingdom; 23 Institute of Human Genetics, University of Leipzig Medical Center, Leipzig, Germany; 24 Division of Neuropaediatrics, Hospital for Children and Adolescents, University Hospital Leipzig, Leipzig, Germany; 25 Department of Neurology, Boston Children’s Hospital, Boston, MA; 26 North West Thames Regional Genomics Service, London North West University Healthcare NHS Trust, Northwick Park Hospital, United Kingdom; 27 Division of Genetics, Department of Pediatrics, East Tennessee State University (ETSU), Quillen College of Medicine, TN; 28 Division of Genetic Medicine, Lausanne University Hospital and University of Lausanne, Lausanne, Switzerland; 29 Speech and Language, Murdoch Children’s Research Institute, Melbourne, Victoria, Australia; 30 Department of Paediatrics, University of Melbourne, Melbourne, Victoria, Australia; 31 Department of Clinical Genetics, Odense University Hospital, Odense, Denmark; 32 Department of Clinical Genetics, Lillebaelt Hospital, location Vejle Hospital, Vejle, Denmark; 33 Department of Mental Health, Odense University Hospital, Odense, Denmark; 34 Department of Clinical Genetics, Leiden University Medical Centre, Leiden, The Netherlands; 35 Division of Genetics and Dysmorphology, Department of Pediatrics, University of California San Diego, Rady Children’s Hospital, San Diego, CA; 36 GeneDx, Gaithersburg, MD; 37 Department of Clinical Genetics, Children’s Health Ireland at Crumlin, Dublin, Ireland; 38 University College Dublin School of Medicine and Medical Science, Dublin, Ireland; 39 Nantes Université, CNRS, INSERM, l’institut du thorax, Nantes, France; 40 CHU Nantes, Service de Génétique Médicale, Nantes Université, CNRS, INSERM, l’institut du thorax, Nantes, France; 41 Department of Clinical Genetics, University Hospitals Bristol and Weston NHS Foundation Trust, Bristol, United Kingdom; 42 Bristol Medical School, University of Bristol, United Kingdom; 43 Institute of Human Genetics, University Medical Center Hamburg-Eppendorf, Hamburg, Germany; 44 Neurology and Neurological Sciences, Division of Child Neurology, Stanford University and Lucile Packard Children’s Hospital, Palo Alto, CA; 45 Division of Genetics, Arnold Palmer Hospital for Children, Orlando, FL; 46 Division of Genetics, Department of Pediatrics, West Virginia University, Morgantown, WV; 47 University of Illinois College of Medicine, Peoria, IL; 48 Exeter Genomics Laboratory, RILD Building, Royal Devon and Exeter NHS Foundation Trust, Exeter, United Kingdom; 49 Geisinger Health System, Danville, PA; 50 University of Arkansas for Medical Sciences, Little Rock, AR; 51 Department of Genetics, Hospital Universitari Germans Trias i Pujol, Catalonia, Spain; 52 Pediatric Neurology Unit, Department of Pediatrics, Hospital Universitari Germans Trias i Pujol, Universitat Autònoma de Barcelona, Catalonia, Spain; 53 Germans Trias i Pujol Research Institute (IGTP), Badalona, Barcelona, Spain; 54 Clinical Institute of Genomic Medicine, University Medical Centre Ljubljana, Ljubljana, Slovenia; 55 Faculty of Medicine, University of Ljubljana, Ljubljana, Slovenia; 56 Department of Clinical Genetics, Leicester Royal Infirmary, University Hospitals of Leicester NHS Trust, Leicester, United Kingdom; 57 Division of Clinical Medicine, University of Sheffield, Sheffield, United Kingdom

**Keywords:** MAP3K enzymes, Neurodevelopmental disorder, *TAOK1*, *TAOK2*, Thousand and one kinase family

## Abstract

**Purpose:**

The thousand and one kinase (TAOK) proteins are a group of serine/threonine-protein kinases involved in signaling pathways, cytoskeleton regulation, and neuronal development. *TAOK1* variants are associated with a neurodevelopmental disorder (NDD) characterized by distinctive facial features, hypotonia, and feeding difficulties. *TAOK2* variants have been reported to be associated with autism and early-onset obesity. However, a distinct *TAOK2*-NDD has not yet been delineated.

**Methods:**

We retrospectively studied the clinical and genetic data of individuals recruited from several centers with *TAOK1* and *TAOK2* variants that were detected through exome and genome sequencing.

**Results:**

We report 50 individuals with *TAOK1* variants with associated phenotypes, including neurodevelopmental abnormalities (100%), macrocephaly (83%), and hypotonia (58%). We report male genital anomalies and hypoglycemia as novel phenotypes. Thirty-seven unique *TAOK1* variants were identified. Most of the missense variants clustered in the protein kinase domain at residues that are intolerant to missense variation. We report 10 individuals with *TAOK2* variants with associated phenotypes, including neurodevelopmental abnormalities (100%), macrocephaly (75%), autism (75%), and obesity (70%).

**Conclusion:**

We describe the largest cohort of *TAOK1*-NDD to date, to our knowledge, expanding its phenotype and genotype spectrum with 30 novel variants. We delineated the phenotype of a novel *TAOK2*-NDD associated with neurodevelopmental abnormalities, autism, macrocephaly, and obesity.

## Introduction

The thousand and one kinase (TAOKs) family is a group of mitogen-activated protein (MAP) kinase kinase kinase (MAP3K) enzymes, which are evolutionarily conserved and ubiquitously expressed in eukaryotes.^1^ They include 3 proteins, TAOK1, TAOK2, and TAOK3, encoded by the *TAOK1* (HGNC:29259), *TAOK2* (HGNC:16835), and *TAOK3* (HGNC:18133) genes, respectively.^2^ They are serine/threonine-protein kinases that act as central regulators in controlling MAPK cascades and thus have roles in signaling pathways, cytoskeleton regulation, neuronal development, and homeostasis.^1,2^ The TAOK family shares a similar domain structure; yet, they differ in amino acid length, including a highly conserved N-terminal catalytic kinase domain, distinct C-terminal domains with a regulatory domain, and 2 to 3 coiled coil (CC) motifs.^1^

The TAOK1 protein has a role in various cellular processes, including neuronal cytoskeleton regulation, DNA damage response, apoptosis regulation, neuronal maturation, and cortical development,^3,4^ mediated via MAPK regulation and stimulation of c-Jun N-terminal kinases (JNK) and p38 MAPK signaling pathways,^5^ and it is highly expressed in the brain.^6^ Monoallelic *TAOK1* pathogenic (P) variants are associated with a neurodevelopmental disorder (NDD) (MIM #610266) characterized by varying degrees of developmental delay (DD), intellectual disability (ID), learning difficulties (LD), distinctive facial features, hypotonia, and feeding and growth difficulties.^4,7^

The TAOK2 protein is implicated in various cellular processes, including neuronal cytoskeleton, microtubule regulation, and synapse development.^8,9^ TAOK2 is a pleiotropic protein and has distinct catalytic kinase and endoplasmic reticulum-microtubule tethering functions.^10^ Altered TAOK2 activity in *Taok2* heterozygous and full knockout (KO) mice is suggested to impair neuronal dendrite growth and synapse development, cause cortical layering abnormalities, and reduce excitatory neurotransmission, through reduction of RhoA activation.^11^ *TAOK2* P variants have been suggested to be a risk factor for autism,^11^ and a potential candidate gene for severe early-onset obesity.^12^ However, a distinct NDD associated with *TAOK2* variants has not been delineated so far.

Here, we characterized 50 previously unreported individuals from 40 families with *TAOK1*-NDD found to have 37 different variants, expanding the phenotype of this condition and reporting 30 novel variants. We also delineated the phenotype and genotype spectrum of *TAOK2* variants in 10 individuals, suggesting an association with a distinct NDD.

## Materials and Methods

The phenotypic and genotypic features of individuals with *TAOK1* and *TAOK2* variants were collected from clinical evaluations across several centers. Individuals were recruited from the Deciphering Developmental Disorders (DDD) study (https://www.ddduk.org/), which contains exome sequencing (ES) data from over 13,000 individuals affected with developmental disorders, individuals identified by GeneDx, as well as through matchmaking on DECIPHER,^13^ GeneMatcher,^14^ and MyGene2.^15^ *TAOK1* and *TAOK2* variants were identified through exome/genome sequencing (either panel-based or agnostic approaches) as trio, duo, or singleton analyses, on a clinical or research basis. Messenger RNA (mRNA) sequencing (on blood-extracted mRNA) was performed for the *TAOK1* c.1909-7G>A intronic variant (details of genetic testing methodologies included in Supplemental Data 1).

### Variant interpretation and computational analysis

Variants were aligned to the human reference genome build (GRCh38/hg38 Assembly) accessed from the National Center for Biotechnology Information database. *TAOK1* variants were annotated using the MANE Select transcript NM_020791.4, and *TAOK2* variants were annotated using the MANE Select NM_016151.4 transcript for TAOK2α isoform and NM_004783.4 for TAOK2β isoform.^16^ Alternative splicing of TAOK2 is known to result in 3 isoforms. Several variants listed in the study fall within exons 1 to 15, which are identical across each isoform; therefore, the specific transcript is not indicated for these variants. The variants (*TAOK2*) NM_016151.4:c.2811dup p.(Cys938LeufsTer56), (*TAOK2*) NM_016151.4:c.2551del p.(Val851CysfsTer45), and the previously reported (*TAOK2*) NM_016151.4:c.2755G>A p.(Asp919Asn) fall within exon 16 in the NM_016151.4 transcript, whereas the variants (TAOK2) NM_004783.4:c.2548delC p.(Arg850GlyfsTer46) and the previously reported NM_004783.4:c.3064_3088del p.(Pro1022Ter) fall within exon 18 and exon 19, respectively, in the NM_004783.4 transcript; therefore, for these specific variants, the corresponding transcript is indicated.

All identified variants were evaluated for pathogenicity and causality according to the American College of Medical Genetics and Genomics/Association for Molecular Pathology classification of sequence variants^17^ and the American College of Medical Genetics and Genomics consensus statement on reporting copy-number variants.^18^ The predicted functional impact of the variants was assessed using in silico prediction tools (CADD, REVEL, AlphaMissense, and SpliceAI). Evolutionary conservation of individual residues was assessed using phyloP (Supplemental Table 1). The variants were checked in PubMed and ClinVar to determine their novelty. The frequency of identified variants was compared with the control population on Genome Aggregation Database (gnomAD v4.1)^19^ and the UK Biobank Allele Frequency data (https://afb.ukbiobank.ac.uk/). The missense variant tolerance at each position in the *TAOK1* and *TAOK2* genes was assessed using Metadome (v1.0.1).^20^ Nonsense-mediated decay (NMD) escape for protein-truncating variants (PTVs) was predicted by the rules described by Lindeboom et al^21^ and aenmd tool.^22^

### In silico protein modeling

Protein modeling was conducted on 11 TAOK1 and 1 TAOK2 residues using DDMut^23^ in wild-type and mutant forms to assess their effect on protein stability and dynamics, and the computed predicted destabilizing effect was indicated by the predicted stability change (ΔΔG Stability _wt → mt_) (kcal/mol).^23^ The residues were modeled on the homologous TAOK2 PDB structure (PDB ID: 2GCD) in *Rattus norvegicus*; the closest structure for TAOK1 with an alignment score of 492 and an identity of 89.4% because of the lack of structural information on the human protein homolog.

### Facial gestalt analysis

DeepGestalt technology was used^24^ via the Face2Gene application (FDNA, Inc) to analyze facial dysmorphology. The facial features of 18 individuals with *TAOK1* variants were compared with a control cohort of unaffected individuals matched for age, sex, and ethnicity. The mean area under the curve (AUC) value represents the degree of discrimination between the cohorts. The mean AUC ranges between 0 and 1 (0 means incorrectly classified cohorts, 0.5 indicates random classifications, and 1 represents perfect discrimination between the cohorts). A *P* value of <.05 indicates that the DeepGestalt technology can distinguish between case and control cohorts.

## Results

We identified 50 individuals (31 males and 19 females) from 40 unrelated families with *TAOK1* variants and 10 individuals (6 males and 4 females) with *TAOK2* variants. Their ages ranged between 2 and 52 years.

### *TAOK1*-NDD cohort phenotypes

**Figure 1:**
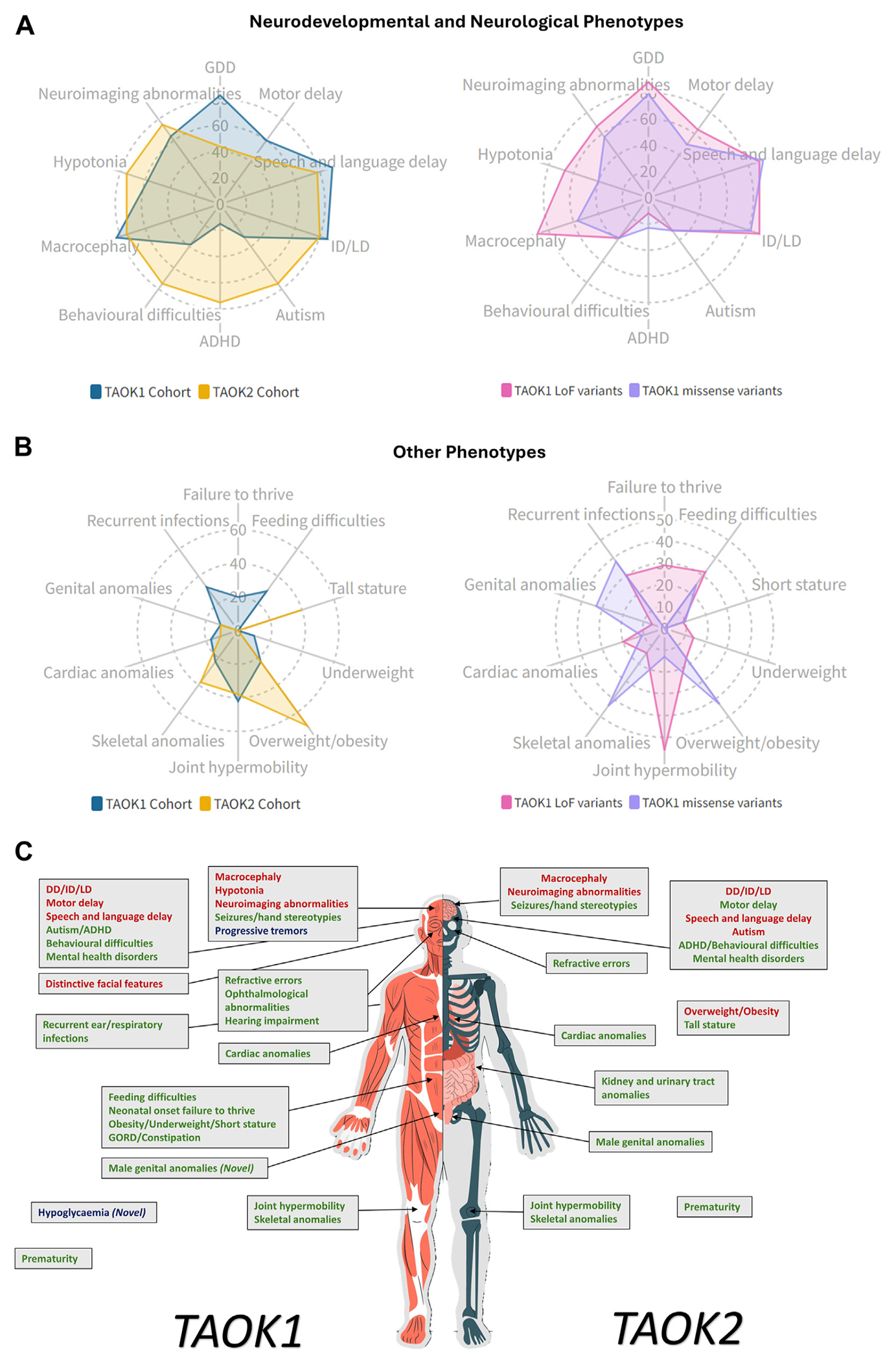
Phenotypic characterisation of individuals with *TAOK1* and *TAOK2* variants in this study. A. A radar chart illustrating the percentage of frequencies of the neurodevelopmental and neurological phenotypes associated with *TAOK1* variants, *TAOK1* LoF variants, *TAOK1* missense variants, and *TAOK2* variants in this study. B. A radar chart illustrating the percentage of frequencies of the other phenotypes associated with *TAOK1* variants, *TAOK1* LoF variants, *TAOK1* missense variants, and *TAOK2* variants in this study. (Figure 1A and B are illustrated by FlourishStudio [https://flourish.studio/]). C. Illustration of the most common phenotypes in individuals with *TAOK1* and *TAOK2* variants in this study. Phenotypes observed in >50% of the study population are highlighted in red, 10% to 50% are highlighted in green, and <10% of the study population are highlighted in blue. (Figure 1C contains elements designed by Freepik, with the original available at www.freepik.com [see details in the Acknowledgments section]). ADHD, attention deficit hyperactivity disorder; DD, developmental delay; GDD, global developmental delay; GORD, gastro-esophageal reflux disorder; ID, intellectual disability; LD, learning difficulties; LoF: loss-of-function.

**Figure 2:**
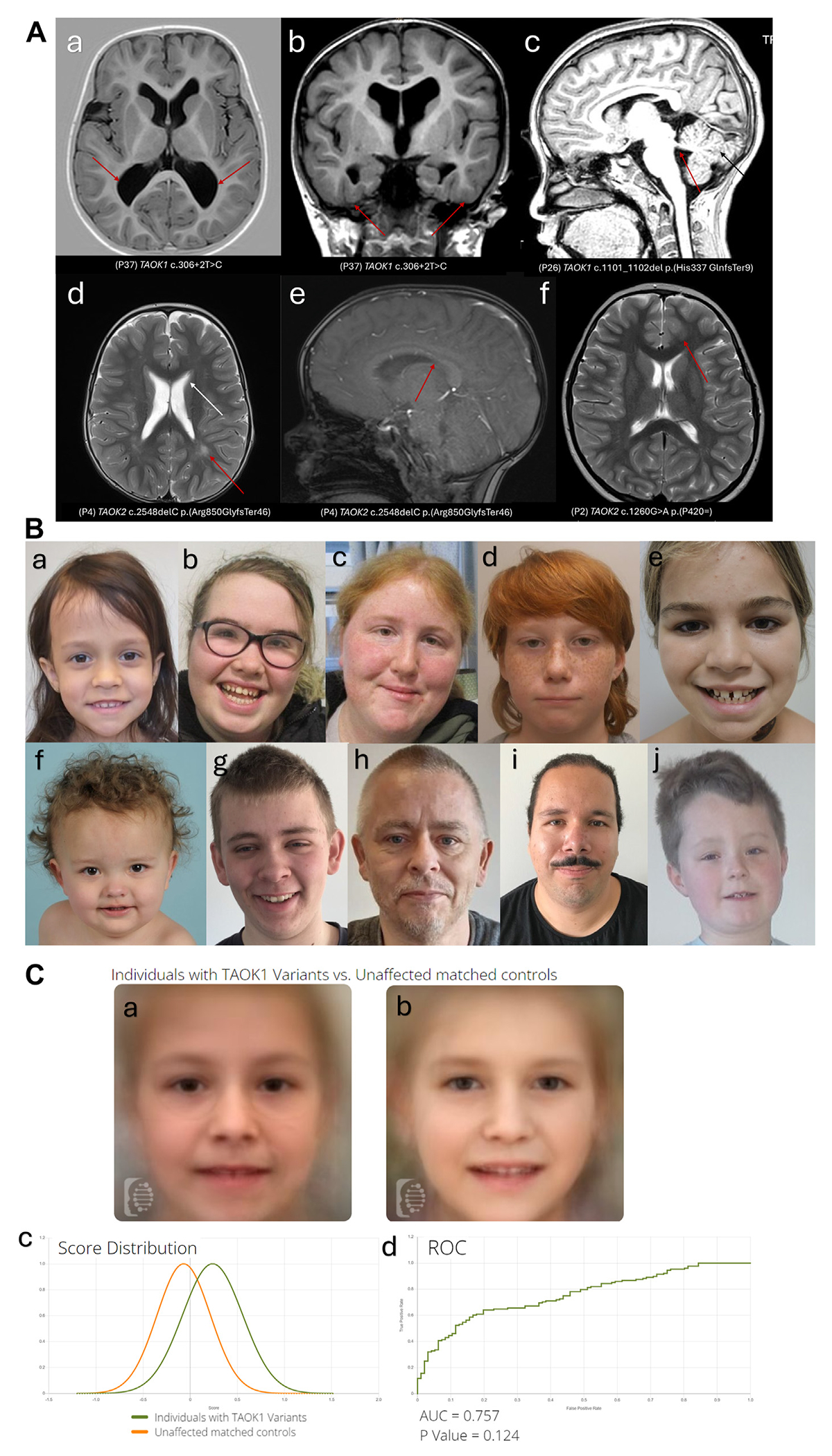
Neuroimaging abnormalities and distinctive facial features in the study cohort. A. a. MRI brain (T1-weighted axial cuts) in patient (P38) with (*TAOK1*) c.306+2T>C variant at age 3.5 years showing ventricular dilatation (red arrows). b. MRI brain (T1-weighted coronal cuts) in the same patient (P38) showing hippocampal malrotation (red arrows). c. MRI brain (T1-weighted axial cuts) in patient (P27) at age 10 years with the (*TAOK1*) c.1102_1103del p.(Val368Ter) variant showing mild hypoplastic cerebellar vermis (red arrow) and mildly elongated superior cerebellar peduncle (black arrow). d. MRI brain (T2-weighted axial cuts) in patient (P4) with (*TAOK2*) NM_004783.4:c.2548del p.(Arg850GlyfsTer46) variant at age 4 years showing focal deep parietal white matter increased signal intensity (red arrow), with a small area of increased signal intensity along the frontal horn of the left lateral ventricle (white arrow). e. MRI brain (T1-weighted sagittal cuts) in the same patient showing mild thinning of the body of the corpus callosum (red arrow). f. MRI brain (T2-weighted axial cuts) in patient (P2) with (*TAOK2*) NM_016151.4:c.1260G>A p.(Pro420=) variant showing left frontal cortical increased signal intensity with blurring of white matter-gray matter junction suggestive of focal cortical dysplasia (red arrow). B. Individual a is (P10). Individuals b (P41) and c (P42) are a proband and her affected mother with. The proband P41 has a de novo *TNRC6B* (HGNC:29190) likely pathogenic variant and a paternally inherited 11q22.3 deletion of uncertain clinical significance. Individual d is (P7). Individual e is (P29). Individual f is (P45). Individuals g (P46) and h (P47) are a proband and his affected father. Individual g has a paternally inherited 1q21.3 duplication of uncertain clinical significance. Individual i is (P40). Individual j (P38) has associated *NOG*-related symphalangism and a *IL1RAPL1* (HGNC:5996) variant of uncertain significance. C. Composite photos were computed from images of individuals with *TAOK1* variants (a) and a control cohort (b). Score distribution (c) and receiver operating characteristic (ROC) (d) curves illustrate the comparison results between individuals with *TAOK1* variants and the control cohort. AUC, area under the curve.

The phenotype findings of individuals with *TAOK1*-NDD in the study were summarized and compared with previously reported findings of 38 individuals in the literature^4,7,25–27^ (Table 1, Figure 1A–C, and Supplemental Table 2). All individuals with *TAOK1* variants presented with neurodevelopmental/neuropsychiatric disorder abnormalities with variable degrees of DD, ID, and LD ranging from borderline to severe motor delay (*n* = 25), speech and language delay (*n* = 36), ID and LD (*n* = 37), autism spectrum disorder (ASD) (*n* = 12), attention deficit hyperactivity disorder (*n* = 6), other behavioral difficulties (*n* = 15), and mental health disorders (including anxiety, depression, and bipolar disorder) (*n* = 6). Affected individuals caught up with motor milestones; yet, speech and language difficulties persisted in 23 individuals assessed between ages 3 and 45 years (including 3 adults). Eighteen individuals required educational support in mainstream education, whereas 9 attended a special school. Developmental regression was observed in 1 individual (P20) who had a second variant of uncertain significance (VUS) in *CDKL5* (HGNC:11411) (seizures were not reported in this individual). Macrocephaly (head circumference more than 2 standard deviations) (*n* = 32/41) and hypotonia (*n* = 18/31) were common. Primary macrocephaly was seen at birth (*n* = 4/11). Seizures and stereotypical hand movements were each reported in 4 individuals, including P36, who has a second diagnosis of *SLC6A1*-related myoclonic-atonic epilepsy (MIM #137165). Neuroimaging abnormalities were observed (*n* = 14/22), with ventriculomegaly being the most common abnormality; other neuroimaging abnormalities included congenital hydrocephalus, cerebral cortical atrophy, cerebral white matter abnormalities, corpus callosum abnormalities, cerebellar atrophy/hypoplasia, brainstem dysplasia, focal cortical dysplasia, and hippocampal abnormalities (Figure 2A).

Neonatal-onset failure to thrive (which subsequently improved in most individuals) (*n* = 6/30) and neonatal-onset feeding difficulties (*n* = 10/34) (which persisted in childhood in 5 individuals with 1 individual requiring a gastrostomy) were reported, and 6 individuals were described as picky eaters well into adolescence, whereas gastrointestinal abnormalities including gastro-esophageal reflux (*n* = 4) and constipation (*n* = 8) were reported. At birth, there were 2 individuals who were small for gestational age (birth weight of less than 10th percentile for gestational age), and 3 were large for gestational age (birth weight of more than 90th percentile for gestational age). Postnatal growth abnormalities were observed in small numbers of individuals with short stature (*n* = 3), underweight (*n* = 3), and overweight/obesity (*n* = 7) (overweight was defined as a body mass index between the 85th and 95th percentile and of 25-29.9 kg/m^2^ in the pediatric and adult individuals, respectively, whereas obesity was defined as body mass index in the 95th percentile or above and of 30 or more kg/m^2^ in the pediatric and adult individuals, respectively).

Joint hypermobility was common (*n* = 11/30), and other skeletal abnormalities were reported in 7 individuals (including scoliosis, pes planus, wrist deformity, valgus calcaneum, splayed ribs, and pes cavus). Individual P38 had symphalangism (attributed to a likely pathogenic [LP] *NOG* (HGNC:7866) variant). Congenital abnormalities were variable and included cardiac abnormalities (*n* = 4) and male genital abnormalities (hypospadias and cryptorchidism) (*n* = 3). Ophthalmological abnormalities (*n* = 17/33) included refractive errors, strabismus, cataract, nystagmus, ptosis, and optic conduction disorder. Recurrent ear/respiratory infections were reported (*n* = 10/31). Eleven individuals were born premature (between 26+3 weeks and 37+3 weeks gestation). Other less common phenotypes included hypoglycemia (transient neonatal or recurrent), hearing impairment, and progressive tremors.

### *TAOK1*-NDD cohort distinctive facial features and facial gestalt analysis

Facial gestalt analysis receiver operating characteristic curves suggested that facial features were distinctive, yet overlapping with the control cohort, with AUC = 0.757 (*P* = .124), suggesting that facial recognition analysis cannot distinguish between *TAOK1*-NDD and the control population (Figure 2C) and that there is no recognizable facial gestalt for *TAOK1*-NDD. However, overlapping distinctive features were reported in 31 individuals, with the most common features being a broad forehead, thin upper vermillion border, hypertelorism, upslanting or down-slanting palpebral fissures, posteriorly rotated ears, low-set ears, high palate, micrognathia, and clinodactyly of the fifth finger (Figure 2B).

### *TAOK1*-NDD cohort genetic findings and phenotyp-genotype correlations

**Figure 3:**
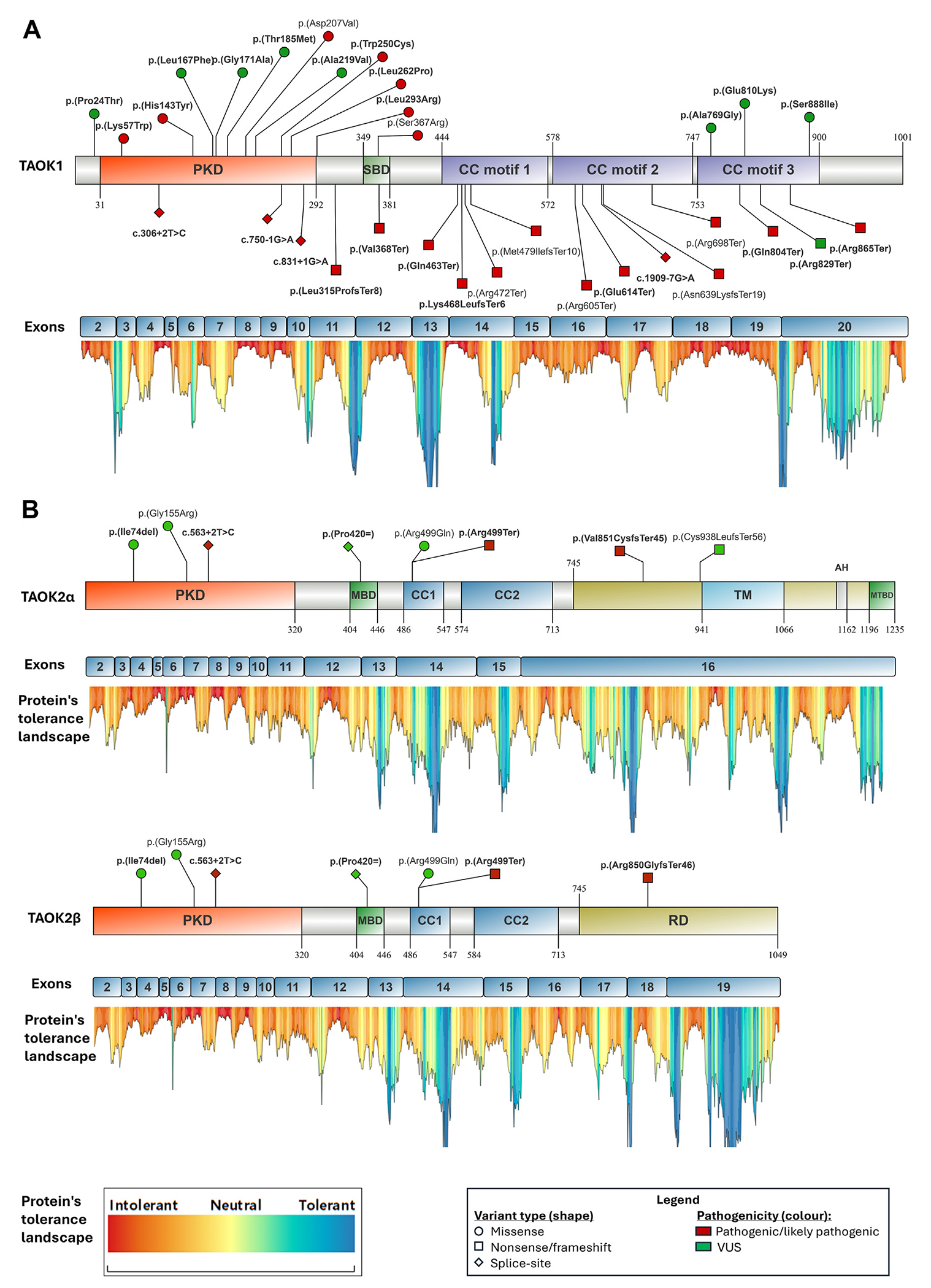
Genotype findings of the cohort of individuals with heterozygous *TAOK1* and *TAOK2* variants. A and B. A schematic representation of the *TAOK1* and *TAOK2* genes, their exons/introns, protein domains, the variants identified, and Metadome’s protein’s tolerance landscape (v1.0.1). Lollipops representing individual variants indicate variant location, classification of (predicted) consequence on the protein (shape), and pathogenicity classification (color). Lollipops representing missense variants are represented on top and other variant types are on bottom. Novel variants are highlighted in bold font. Larger deletions (individuals P4, P5, P34, P40, and P49) are not demonstrated. The Metadome protein tolerance landscape is shown color-coded below the protein scheme and illustrates that *TAOK1* missense variants (in and outside the protein kinase domain) are associated with regions of high intolerance to protein variation. The figure is illustrated by IBS 2.0.^28^ AH, amphipathic helix; CC, coiled-coil motif; MBD, MEK binding domain; MTBD, microtubule binding domain; PKD, protein kinase domain; RD, regulatory domain; SBD, substrate-binding domain; TM, transmembrane domains; VUS, variant of uncertain significance.

The genotype findings of individuals with *TAOK1*-NDD in the study were detailed (Figure 3A, Supplemental Table 1). Thirty-seven monoallelic *TAOK1* variants were identified, 30 of which were not previously reported. Variant types included missense (*n* = 17), stop-gain (*n* = 9), frameshift indels (*n* = 5), splice-site/intronic (*n* = 4) and multi-exon deletions (*n* = 4) (3 intragenic deletions of exons 9 and 10, exons 1-8, exon 16-20, and the fourth deletion encompassing the N terminus, including the promoter, exon 1, and part of exon 2). Six variants were predicted to have substantial splicing effects by in silico tools. mRNA sequencing for *(TAOK1)* c.1909-7G>A variant showed aberrant splicing with activation of a cryptic splice-site resulting in intron inclusion of 5 bp, creating a frameshift, and the transcript is predicted to undergo NMD (Supplemental Figure 1). The variants were de novo in 15 individuals, inherited from an affected parent in 11 individuals, whereas inheritance was unknown in 24 individuals. The variants were absent or observed at extremely low allele frequencies across gnomAD (v4.1) and UK Biobank control populations. All of the missense variants involved conserved residues, most of them were associated with residues of high intolerance to missense variation on Metadome and *n* = 11 of 17 clustered in the protein kinase domain (residues 34-295) (Figure 3A, Supplemental Table 1). In silico protein modeling predictions suggest that the 11 modeled *TAOK1* missense variants have variable degrees of predicted decreased or increased intramolecular stability effects on the protein. The *(TAOK1)* c.70C>A p.(Pro24Thr), c.170A>C p.(Lys57Thr), c.785T>C p.(Leu262Pro), and c.878T>G p.(Leu293Arg) residues affecting the PK domain were associated with the most predicted destabilizing effects (Supplemental Table 1, Supplemental Figure 2). The most frequent variants were the previously reported *(TAOK1)* c.2092C>T p.(Arg698Ter), the novel c.1414C>T p.(Arg472Ter) and c.2485C>T p.(Arg829Ter) variants, seen in 2 families each, whereas most of the *TAOK1* variants were not recurrent. Twenty-eight variants were classified as either P or LP, whereas 10 variants were classified as VUS.

Twenty individuals had additional genetic findings, including P/LP variants (dual genetic diagnosis) and VUS, in 11 copy-number variants and single-nucleotide variants in 21 genes. The confirmed second genetic diagnoses (P/LP variants) included 17q11.2 deletion (including *NF1* (HGNC:7765) (P39), NOG (HGNC:7866)-related symphalangism (P38), *TNRC6B* (HGNC:29190)-NDD (P41), *SLC6A1* (HGNC:11042)-related myoclonic-atonic epilepsy (P36), congenital adrenal hyperplasia (21-hydroxylase deficiency) (P35), and 16p13.11 duplication (P11, P12) (Supplemental Table 1). These additional findings may contribute to the phenotypes in the study and overlap with *TAOK1*-NDD phenotypes of the respective individuals (Table 1, ^4,7,25–27^ Supplemental Table 2). Phenotypic features of individuals with loss-of-function (LoF) variants (including stop-gain, frameshift, deletions, splicing variants, as well as missense variants predicted to affect splicing) were compared with those with missense variants (Table 1 ^4,7,25–27^). None of the individuals with missense variants presented with seizures or neonatal failure to thrive. Genital anomalies (33% vs 6%) and overweight/obesity (43% vs 17%) were more common in individuals with missense variants; yet, macrocephaly (57% vs 89%) and hypotonia (40% vs 67%) were less common in them compared with the other group.

### *TAOK2*-NDD cohort phenotypes

The phenotype findings of individuals with *TAOK2* variants in the study were summarized (Table 1 ^4,7,25–27^ and Figure 1) and described in detail (Table 2, ^29^ Supplemental Table 2). All individuals (*n* = 10) with *TAOK2* variants presented with neurodevelopmental abnormalities (100%) with variable degrees of DD, ID, and LD ranging from borderline to severe (motor delay [*n* = 4], speech and language delay [*n* = 7], ID/LD [*n* = 8], ASD [*n* = 6], attention deficit hyperactivity disorder, and other behavioral difficulties [*n* = 2], and mental health disorders [*n* = 1]). Speech and language difficulties persisted in all 7 individuals assessed between ages 3 and 7.7 years. One individual (P3) presented with childhood apraxia of speech. Macrocephaly was common (*n* = 6). Abnormal brain neuroimaging (MRI) (*n* = 2/3) included cortical migration abnormalities, thin corpus callosum, and white matter abnormalities (Figure 2A). Growth abnormalities, including overweight/obesity (*n* = 7) and tall stature (*n* = 4) were common. Skeletal abnormalities (*n* = 3) included scoliosis, arachnodactyly, pes planus, and joint hypermobility. Congenital abnormalities were rare and included retractile testes, unilateral microtia, preauricular tag, unilateral small kidney, bladder diverticulum, and bicuspid aortic valve. There was no recognizable facial gestalt, with few distinctive features in 2 individuals.

### *TAOK2*-NDD cohort genetic findings

The genotype findings of individuals with *TAOK2* variants in the study were detailed (Table 2, ^29^ Figure 3B, and Supplemental Table 2). We identified 6 novel and 3 previously reported monoallelic *TAOK2* variants, which included 3 frameshift indels present in either of TAOK2α (*n* = 2) or TAOK2β isoforms (*n* = 1), missense (*n* = 2), splice-site (*n* = 2), stop-gain (*n* = 1), and inframe deletion (*n* = 1) present in both TAOK2α and TAOK2β isoforms. The variants were de novo in 4 individuals, inherited from an affected parent in 2 individuals, and had unknown inheritance in 4 individuals. Four variants were classified as P/LP and 5 as VUS, assuming an established *TAOK2* disease-gene association. The 3 *TAOK2* frameshift indels in the last exon (located in the regulatory domain) are predicted to escape NMD, resulting in a truncated TAOK2α or TAOK2β protein (C-terminal deletion). The *TAOK2* c.563+2T>C variant is predicted by in silico tools to abrogate intron 7 donor splice-site resulting in intron 7 retention. The *(TAOK2)* c.1260G>A, predicted as a synonymous *(TAOK2)* c.1260G>A p.(Pro420=) variant, affects a canonical splice-site and is likely to result in donor splice-site loss, although splicing studies have not been performed for both variants. The *(TAOK2)* c.1496G>A p.(Arg499Ter) affects both isoforms and therefore likely results in haploinsufficiency. The *(TAOK2)* c.221_223del p.(Ile74del) and c.463G>A p.(Gly155Arg) variants, both located in the kinase domain, affect residues that are evolutionarily conserved and intolerant to missense variation. The *(TAOK2)* c.1496G>A p.(Arg499Gln) variant, located in the CC motif 1 similarly affects a conserved residue. One individual (P10) had an additional genetic finding (a monoallelic *CELF2* (HGNC:2550) c.1456 G>A VUS).

## Discussion

Here, we characterized the clinical and genetic findings in 50 individuals from 40 families with *TAOK1*-NDD harboring 37 *TAOK1* variants, reporting 30 novel variants, and expanding the phenotype of *TAOK1*-NDD with novel phenotype-genotype associations. We expanded the phenotype of *TAOK1*-NDD to include transient neonatal and recurrent hypoglycemia and male genital abnormalities, which have not been previously reported to our knowledge. TAOKs have a role in the activation of the p38 MAPK pathway because they function as MAP3Ks, which phosphorylate MKK3/6 with the subsequent phosphorylation of p38 kinases.^1^ p38 MAPK has a crucial role in glucose homeostasis,^30,31^ and p38 MAPK inhibition was found to exert a hypoglycemic effect in db/db mice (an animal model of type 2 diabetes) by improving β-cell function via inhibition of β-cell apoptosis.^32^ We report significant inter- and intrafamilial phenotypic variability with variable penetrance because there are several individuals who have inherited the *TAOK1* variants from mildly affected parents with mild ID or LD, which was also observed in previous studies.^4^ This is supported by the presence of LoF *TAOK1* variants in the control population on gnomAD with extremely low allele frequencies, suggesting possible reduced penetrance and that *TAOK1*-NDD could be an underdiagnosed condition. We observed dual genetic diagnoses with *TAOK1*-NDD and a second genetic disorder, with overlapping phenotypes in some individuals, in 14% of individuals with *TAOK1* variants, relatively higher than previous reports.^33,34^ This is suggested to be due to the cohort being enriched for neurodevelopmental abnormalities and highlights the complexities in the genetic investigation of individuals with NDDs and phenotyping affected individuals with atypical clinical features. The presence of dual genetic diagnoses and genetic findings classified as VUS were also observed in previous reports of *TAOK1*-NDD.^4,7^

We observed that most of the *TAOK1* missense variants in the study clustered at the protein kinase domain, similar to previous reports,^4,7^ at residues with high intolerance to missense variation.^20^ The kinase domain is structurally evolutionarily conserved^35^ and is highly constrained for missense variation (*z* = 5.92),^19^ with the N terminus, which includes part of the kinase domain, being significantly constrained to missense variation (*P* = 1.05 × 10^−7^).^35^ The *(TAOK1)* c.332C>T p.(Ser111Phe), c.500T>G p.(Leu167Arg), c.656C>T p.(Ala219Val), and c.806G>A p.(Arg269Gln) variants located in the kinase domain were previously found by in vitro kinase assays to affect the catalytic function of the kinase domain.^35^ The kinase activity was found to be crucial for TAOK1 subcellular localization and membrane architecture.^35^ The *(TAOK1)* c.449G>T p.(Arg150Ile) variant was found to reduce the kinase activity, whereas the *(TAOK1)* c.500T>G p.(Leu167Arg) was found to act in a dominant-negative manner.^4^ This supports that missense variants, especially in the kinase domain, are a mechanism of *TAOK1*-NDD, in addition to haploinsufficiency, which is an established mechanism of this disorder.^4,7^ It was found that *Taok1* haploinsufficiency in mice models resulted in macrocephaly, autistic-like behaviors, and neuronal abnormalities in the dorsal raphe nucleus (DRN).^36^ Genetic deletion of Taok1 in VGlut3-positive neurons of DRN resulted in autistic-like behaviors in adult mice models, which were reversed by the reintroduction of wild-type Taok1 (but not kinase-dead Taok1) into the DRN. This supports the observations of macrocephaly and autism as common phenotypes with *TAOK1*-NDD and highlights the importance of the TAOK1 kinase activity in the neurons.^7^ We report the novel *(TAOK1)* c.2593C>T p.(Arg865Ter) in exon 20, which is predicted to escape NMD and result in a truncated protein (loss of the terminal 137 amino acids, including the C-terminal part of the CC motif 2). The CC motifs in TAOK1 are predicted to fold into a triple helix and are important for plasma membrane tubulation through phospholipid binding.^35^ In vitro studies found that the C-terminal tail (residues 901-1001) was dispensable; yet, the residues 321-901 were necessary and sufficient for TAOK1 plasma membrane association and membrane protrusion generation.^35^ Therefore, this variant may impair TAOK1 plasma membrane localization. We also observed that none of the variants in our study or those reported in the literature with *TAOK1*-NDD were located in the C-terminal tail, supporting the studies suggesting that it is functionally dispensable.

We outlined the phenotypic spectrum of *TAOK2*-NDD in 10 individuals harboring 9 heterozygous *TAOK2* variants, reporting 6 novel variants. Our findings suggest that *TAOK2* variants are associated with neurodevelopmental abnormalities, autism, macrocephaly, obesity, and variable congenital abnormalities, suggesting a novel disease-gene association. TAOK2 protein is highly expressed during neuronal development,^37^ with distinct catalytic kinase and endoplasmic reticulum-microtubule tethering functions,^10^ and is important for neuronal development and function, dendrite development,^10,38^ and synapse development.^11,39^ TAOK2 was found to act as a translational repressor through interaction with eukaryotic elongation factor 2 to modulate phosphorylation on key regulatory sites. This requires the kinase activity of TAOK2 and is independent of eukaryotic elongation factor 2 kinase activity.^40^ *TAOK2* is located in the 16p11.2 region, and 16p11.2 deletions were reported to be associated with neurodevelopmental abnormalities, childhood apraxia of speech, epilepsy, and obesity with incomplete penetrance and clinical heterogeneity.^41–45^ The reciprocal 16p11.2 duplications were found to be associated with susceptibility to autism and schizophrenia.^41,43^ Both LoF and missense *TAOK2* variants were previously reported in association with autism,^11,46^ suggesting that it is a critical gene in the locus.

The brains of the *Taok2* KO and 16p11.2 heterozygous deletion mouse models were found to show similar abnormalities, with reduced levels of phosphorylated JNK1 and cortical neuronal abnormalities. Taok2 rescued autism-related developmental abnormalities in a 16p11.2 heterozygous deletion mouse model, which further highlighted the association of *TAOK2* variants with NDDs, including ASD.^46^ *Taok2* heterozygous and KO mice were also found to have neural connectivity, cortical layering, dendrite and synapse formation abnormalities, and decreased excitatory neurotransmission,^11^ as well as dosage-dependent increased brain size yet with decreased volumes of the corpus callosum, anterior commissure, and olfactory bulbs.^11^ Taok2 overexpression and downregulation were found to disrupt neuronal migration.^47^ Furthermore, the 16p11.2 heterozygous deletion mouse model demonstrated in vivo cerebral cortical translational abnormalities with increased cortical neuronal protein synthesis, which was rescued with TAOK2 or TAOK2β reintroduction.^40^ Alternative splicing of *TAOK2* results in 3 isoforms, with TAOK2α and TAOK2β sharing exons 1 to 16. TAOK2α is the longest isoform and TAOK2β has a distinct C terminus from alternative splicing of alternate exons at the 3′ end. TAOK2γ is a shorter isoform that is alternatively spliced at the 3′ end; yet, it maintains the reading frame.^9^ Neuronal positioning during cortical development was found to be affected in an isoform-specific manner with TAOK2α variants but not TAOK2β variants, causing abnormal neuronal migration as the TAOK2α isoform colocalizes with microtubules and activates JNK1.^47^ JNK1 is involved in neuronal differentiation and microtubule dynamics.^48^ TAOK2β was found to affect synapse morphology and connectivity through RhoA-dependent actin modulation.^47^ Therefore, it was proposed that the 2 isoforms have distinct functional roles, with TAOK2α modulating early developmental processes, such as neuronal migration, by affecting microtubules and TAOK2β being involved in developmental processes that occur later, such as neuronal differentiation and synaptic connectivity, by affecting the actin cytoskeleton.^47^ These findings were similar to what was observed in the *TAOK2* study cohort, with macrocephaly being a common feature and similar neuroimaging abnormalities. However, neuroimaging was performed in only a few individuals in our study, and volumetric brain imaging to estimate brain volume abnormalities would be of interest.

*TAOK2* was suggested as a potential candidate gene for human obesity through the analysis of ES data in individuals with severe early-onset obesity, with the transmembrane domains (amino acids 941-1162),^12^ which is required for the localization of TAOK2 to ER subdomains,^10^ being enriched for very rare variants associated with obesity.^12^ TAOK2 was identified as a critical modulator of the Hippo signaling pathway through direct phosphorylation,^12,49^ and knockdown of Drosophila *tao* increased adiposity and triglyceride levels in vivo in the *Drosophila* ortholog of TAOK2.^12^ The Hippo signaling pathway has a role in regulation of cell proliferation and growth and organ size,^50^ and its upregulation is associated with obesity and increased adiposity.^51^ Growth abnormalities, including obesity and/or tall stature, were observed in most individuals with *TAOK2* variants in our study, particularly in those with variants that are not located in the C-terminal transmembrane domains.

We report several *TAOK2* variants, including missense, truncating indels, and splice-site variants. *TAOK2* is highly constrained to LoF variation (pLI = 1) and missense variation (*z* = 3.36),^19^ suggesting that both missense and LoF *TAOK2* variants could be deleterious.^11^ We report 3 *TAOK2* frameshift indels in the last exon that are predicted to cause a truncated protein (C-terminal deletion) in the TAOK2α isoform in 2 individuals, and the TAOK2β isoform in the third. The C terminus of TAOK2 is a negative regulator of TAOK2 kinase activity^52,53^ and is required for microtubule binding and stabilization independently of kinase activity.^54^ The truncating *(TAOK2)* NM_004783.4:c.3064_3088del p.(Pro1022Ter) variant (affecting the TAOK2β isoform only) was previously found by functional studies to result in gain of function with TAOK2 overexpression, increased kinase activity, and impaired protein stability; yet, it did not affect auto-phosphorylation.^11^ The missense *(TAOK2)* NM_016151.4:c.2755G>A p.(Asp919Asn) in the regulatory domain was found to have no effect on kinase activity, nor on JNK activation, yet severely reduced nuclear localization of TAOK2 and apoptotic membrane blebbing.^55^ Functional studies have not been performed for the frameshift indels in our study, but our hypothesis is that this is likely to result in gain of function, similar to *(TAOK2)* NM_004783.4:c.3064_3088del p.(Pro1022Ter) because of the truncation of TAOK2 C-terminal regulatory domain.

The *(TAOK2)* c.221_223del p.(Ile74del) and c.463G>A p.(Gly155Arg) variants are reported, and they affect residues intolerant to missense variation in the kinase domain. Another missense variant located in the kinase domain *(TAOK2)* c.403G>C p.(Ala135Pro) (present in both TAOK2α and TAOK2β isoforms) was previously found by in vitro to impair auto-phosphorylation and reduce TAOK2 kinase function with resultant LoF^11^ and impaired translational repression function.^40^ The *(TAOK2)* c.506A>C p.(Asp169Ala) variant was found by in vitro studies to cause loss of kinase activity, yet not affecting MAP3K7-mediated activation of NF-kappa-B or interaction with TAOK1.^56^ Functional studies have not been performed for the *(TAOK2)* c.221_223del p.(Ile74del) variant in our study, but our hypothesis is that it may impair the kinase domain function. We report the *(TAOK2)* c.563+2T>C variant, which is predicted by in silico tools to result in a splice donor site loss and intron 7 retention. The *(TAOK2)* c.563+12_563+15del variant affecting the same splice donor site has been previously found to result in intron 7 retention and introduction of a premature stop codon on RNA expression studies.^11^ Both LoF and truncating *TAOK2* variants are observed in the control population on gnomAD with low allele frequencies, suggesting possible reduced penetrance.

The *TAOK3* (HGNC:18133) gene encodes thousand and one amino acid kinase 3,^57^ which has a similar role as a regulator of MAPK cascades yet with distinctive functions of ERKs and p38 MAPK activation and JNK inhibition.^2^ Previous large-scale ES and genome-wide association studies^58–60^ suggest that *TAOK3* variants may be associated with an increased risk of neurodevelopmental abnormalities and mental health disorders; yet, a distinct *TAOK3*-related disorder has not been delineated. Our preliminary findings of *n* = 12 LoF and *n* = 10 missense variants suggest that *TAOK3* variants are associated with a distinct neurodevelopmental disorder with developmental delay, intellectual disability, microcephaly, and seizures (unpublished data).

In conclusion, we describe the largest cohort of *TAOK1*-NDD published to date. We expand the phenotypic spectrum of *TAOK1*-NDD to include male genital anomalies and hypoglycemia and expand the genotypes of *TAOK1*-NDD with 30 novel variants. Our findings confirm the previous observations that the *TAOK1* kinase domain is a hotspot for missense variation. We delineated the phenotypic characteristics of a potential novel *TAOK2*-NDD that is associated with neurodevelopmental abnormalities, autism, macrocephaly, and obesity, which support the previous observations that *TAOK2* missense and truncating variants are associated with human disease. Both *TAOK1*- and *TAOK2*-NDDs share overlapping phenotypes with neurodevelopmental abnormalities and macrocephaly; yet, feeding difficulties and congenital abnormalities are more common with *TAOK1*-NDDs, and obesity is more common with *TAOK2*-NDDs.

## Supplementary Material

supplemental material

The online version of this article (https://doi.org/10.1016/j.gim.2024.101348) contains supplemental material, which is available to authorized users.

## Figures and Tables

**Table 1:** Summary of the phenotypic data of the study population compared with previously reported individuals with *TAOK1* variants in literature

Study Population	*TAOK1* Study Population (*n* = 50)	*TAOK1* LoF/Truncating Variants (*n* = 34)^a^	*TAOK1* Missense Variants (*n* = 16)	*TAOK1-NDD* Previously Reported Cases in Literature (*n* = 38)^4,7,25–27^	*TAOK2* Study Population (*n* = 10)
**1. Demographic data**					
Sex: male/female, *n* (%)	31/19 (62%/38%)	19/14 (58%/42%)	12/4 (75%/25%)	22/16 (58%/42%)	6/4 (60%/40%
Age at last assessment (median-range) (years)	11.1 (2.17-52)	10 (3-43)	12.2 (2.2-52)	5 (1.2-21)	6.8 (2-24)
**2. Neurodevelopmental phenotypes, *n* (%)**					
Global developmental delay	33/40 (83%)^b^	22/25 (88%)	11/14 (79%)	29/34 (85%)	4/9 (44%)
Severe	6/40 (15%)	3/25 (12%)	3/14 (21%)	N/A	1/9 (11%)
Mild/Moderate	23/40 (58%)	17/25 (68%)	6/14 (43%)	N/A	3/9 (33%)
Nonspecified	5/40 (13%)	2/25 (8%)	2/14 (14%)	N/A	0/9 (0%)
Motor delay	25/42 (60%)	18/28 (64%)	7/14 (50%)	10/14 (71%)	4/9 (44%)
Speech and language delay	36/40 (90%)	24/27 (89%)	12/13 (92%)	10/14 (71%)	7/9 (78%)
Persistent speech and language difficulties	23/32 (72%)	15/21 (71%)	8/11 (73%)	N/A	7/9 (78%)
Intellectual disability/learning difficulties	37/43 (86%)	24/27 (89%)	13/16 (82%)	19/30 (63%)	8/10 (80%)
Mainstream education (without support)	3/34 (9%)	3/23 (13%)	0/11 (0%)	N/A	1/4 (25%)
Mainstream education (with support)	18/34 (53%)	12/23 (52%)	6/11 (55%)	N/A	3/4 (75%)
Special education	9/34 (27%)	6/23 (26%)	3/11 (27%)	N/A	0/4 (0%)
Educational support non-specified	4/34 (12%)	2/23 (9%)	2/11 (18%)	N/A	0/4 (0%)
Autistic spectrum disorder/traits	12/39 (31%)	8/26 (31%)	4/13 (31%)	8/35 (24%)	6/8 (75%)
ADHD	6/39 (15%)	3/26 (12%)	3/13 (23%)	19/35 (56%)	2/8 (25%)
Behavioral difficulties	15/39 (38%)	10/26 (38%)	5/13 (38%)	14/27 (52%)	2/8 (25%)
Mental health disorders	6/32 (19%)	3/21 (14%)	3/11 (27%)	4/25 (16%)	1/6 (17%)
**3. Neurological phenotypes, *n* (%)**					
Macrocephaly	32/41 (83%)	24/27 (89%)	8/14 (57%)	18/33 (55%)	6/8 (75%)
Absolute macrocephaly	25/41 (64%)	18/27 (67%)	7/14 (50%)	14/33 (42/%)	4/8 (50%)
Relative macrocephaly	7/41 (19%)	6/27 (22%)	1/14 (7%)	4/33 (12%)	2/8 (25%)
Primary macrocephaly (at birth)	4/14 (29%)	4/11 (36%)	0/3 (0%)	N/A	0/3 (0%)
Hypotonia	18/31 (58%)	14/21 (67%)	4/10 (40%)	20/33 (61%)	0/9 (0%)
Seizures	4/50 (8%)^c^	4/34 (12%)^c^	0/16 (0%)	2/13 (15%)	1/9 (11%)
Abnormal movements/hand stereotypies	5/50 (10%)	3/34 (9%)	2/16 (12%)	2/38 (5%)	1/9 (11%)
Neuroimaging abnormalities	14/22 (64%)	10/15 (67%)	4/7 (57%)	11/30 (37%)	2/3 (67%)
Ventriculomegaly/hydrocephalus	4/22 (18%)	3/15 (20%)	1/7 (14%)	7/21 (33%)	0/10 (0%)
**4. Growth/gastrointestinal phenotypes, *n* (%)**					
Neonatal-onset failure to thrive	6/30 (20%)	6/21 (29%)	0/9 (0%)	3/20 (15%)	0/10 (0%)
Feeding difficulties	10/34 (29%)	7/22 (32%)	3/12 (25%)	13/23 (57%)	0/10 (0%)
Small for gestational age	2/30 (7%)	1/22 (5%)	1/8 (13%)	1/24 (4%)	0/7 (0%)
Large for gestational age	3/30 (10%)	3/22 (14%)	0/8 (0%)	3/24 (13%)	2/7 (29%)
Short stature (height for age <2.0 SD)	3/32 (9%)	2/22 (9%)	1/10 (10%)	4/20 (20%)	0/10 (0%)
Tall stature (height for age >2.0 SD)	0/32 (0%)	0/22 (0%)	0/10 (0%)	1/20 (5%)	4/10 (40%)
Underweight	3/30 (10%)	3/22 (14%)	0/7 (0%)	5/28 (18%)	0/10 (0%)
Overweight/obesity (weight for height >2.0 SD)	7/30 (23%)^d^	4/23 (17%)	3/7 (43%)	10/28 (36%)	7/10 (70%)
Gastro-esophageal reflux	4/26 (15%)	2/17 (12%)	2/9 (22%)	6/27 (22%)	0/10 (0%)
Constipation	8/30 (27%)	4/20 (20%)	4/10 (40%)	5/24 (21%)	0/10 (0%)
**5. Skeletal phenotypes, *n* (%)**					
Joint hypermobility	11/30 (42%)	10/21 (56%)	1/9 (13%)	12/32 (38%)	3/8 (38%)
Skeletal anomalies	7/30 (23%)^e^	3/21 (14%)	4/9 (44%)^e^	N/A	3/8 (38%)
**6. Other phenotypic abnormalities and congenital anomalies, *n* (%)**					
Distinctive facial features	31/46 (70%)	22/32 (67%)	9/14 (64%)	15/20 (75%)	2/8 (25%)
Refractive errors	11/33 (33%)	8/22 (36%)	3/11 (27%)	1/8 (13%)	2/6 (33%)
Other ophthalmological abnormalities	11/33 (33%)	6/22 (27%)	5/11 (46%)	1/8 (13%)	0/10 (0%)
Hearing impairment	4/34 (12%)	3/24 (13%)	1/10 (10%)	1/8 (13%)	0/10 (0%)
Cardiac anomalies	4/24 (17%)	3/15 (20%)	1/9 (11%)	3/15 (20%)	1/9 (11%)
Kidney and urinary tract anomalies	0/25 (0%)	0/16 (0%)	0/9 (0%)	3/35 (9%)	1/9 (11%)
Genital anomalies	3/28 (11%)	0/19 (6%)	3/9 (33%)	N/A	1/9 (11%)
Prematurity	11/39 (28%)	6/25 (24%)	5/14 (36%)	3/19 (16%)	2/9 (22%)
Polyhydramnios	0/50 (0%)	0/34 (0%)	0/16 (0%)	5/25 (20%)	0/10 (0%)
Recurrent ear/respiratory infections	10/31 (32%)	7/23 (30%)	3/8 (38%)	6/18 (33%)	0/10 (0%)
Hypoglycemia	3/50 (6%)	3/34 (9%)	0/16 (0%)	0/38 (0%)	0/10 (0%)

*ADHD*, attention deficit hyperactivity disorder; *LoF*, loss-of-function; *N/A*, not available; *NDD*, neurodevelopmental disorder; *VUS*, variant of uncertain significance.

a Including stop-gain, frameshift, deletions, splicing variants, and missense variants predicted to affect splicing.

b Including P39 with neurofibromatosis type 1 (17q11.2 deletion including *NF1*), P41 with *TNRC6B*-related neurodevelopmental disorder, P11 and P12 with 16p13.11 duplication.

c Including P36, who has a second diagnosis of *SLC6A1*-related myoclonic-atonic epilepsy. Seizures were not reported in P20 who had a second VUS in *CDKL5*.

d Including P11 with 16p13.11 duplication. Data on body weight is not available for P12 with 16p13.11 duplication.

e Excluding P38, who had symphalangism (fusion of proximal interphalangeal joints) (attributed to a likely pathogenic NOG variant).

**Table 2:** Details of the phenotype and genotype findings of individuals with *TAOK2* variants in the study

Study Participants	P1	P2	P3^a^	P4	P5	P6	P7	P8	P9 (Mother of P8)	P10
Neurodevelopmental and neurological phenotypes	NDD, ID, ASD Macrocephaly	Speech and language delay, epilepsy hallucinations	NDD, mild ID, speech and language delay (childhood apraxia of speech), autistic features macrocephaly	NDD, ID, ASD macrocephaly	NDD, macrocephaly	NDD, ID, ASD Relative macrocephaly	Speech and language delay	Speech and language delay, ID, ASD, ADHD, behavioral difficulties, macrocephaly	ID, learning difficulties	ASD, ADHD, LD, behavioral difficulties
Growth abnormalities	Obesity, tall stature	Obesity	Obesity	Obesity, tall stature	LGA tall stature	N/A	Obesity	Obesity	N/A	Obesity, tall stature
Neuroimaging	N/A	Neuronal migration abnormalities	N/A	Thin corpus callosum deep focal WM signal abnormalities	N/A	N/A	N/A	N/A	N/A	
Other phenotypes	Prematurity Joint hypermobility arachnodactyly refractive errors retractile testes	Chronic diarrhea	N/A	Joint hypermobility pes planus Refractive errors Unilateral ear tag, high forehead, hirsutism, clinodactyly	Scoliosis	N/A	Unilateral microtia bicuspid aortic valve	Bladder diverticulum	Death of stomach cancer (age 24)	Juvenile idiopathic scoliosis, joint hypermobility, premature pubarche
HGVS genomic variant description	NC_000016.10:g. 29979310T>C	NC_000016.10:g. 29983332G>A	NC_000016.10:g. 29985286G>A	NC_000016.10:g. 29990886del	NC_000016.10:g. 29978268_29978270del	NC_000016.10:g. 29987083dup	NC_000016.10:g. 29986823del	NC_000016.10:g. 29985285C>T	NC_000016.10:g. 29985285C>T	NC_000016.10:g. 29979208G>A
Variant (nucleotide change and protein change)^b^	NM_016151.4:c. 563+2T>C NM_004783.4:c. 563+2T>C	NM_016151.4:c. 1260G>A p.(Pro420=) NM_004783.4:c. 1260G>A p.(Pro420=)	NM_016151.4:c. 1496G>A p.(Arg499Gln) NM_004783.4:c. 1496G>A p.(Arg499Gln)	NM_016151.4:c. *2906del NM_004783.4:c. 2548delC p. (Arg850GlyfsTer46)	NM_016151.4:c. 221_223del p.(Ile74del) NM_004783.4:c. 221_223del p.(Ile74del)	NM_016151.4:c. 2811dup p. (Cys938LeufsTer56) NM_004783.4:c. 2232+579dup	NM_016151.4:c. 2551del p. (Val851CysfsTer45) NM_004783.4:c. 2232+319del	NM_016151.4:c. 1495C>T (p.Arg499Ter) NM_004783.4: c.1495C>T (p.Arg499Ter)	NM_016151.4:c. 1495C>T (p.Arg499Ter) NM_004783.4:c. 1495C>T (p.Arg499Ter)	NM_016151.4:c. 463G>A p.(Gly155Arg) NM_004783.4:c. 463G>A p.(Gly155Arg)
TAOK2α isoform variant type	Splice-site	Synonymous	Missense	Intronic	Inframe indel	Frameshift indel	Frameshift indel	Nonsense	Nonsense	Missense
TAOK2β isoform variant type	Splice-site	Synonymous	Missense	Frameshift indel	Inframe indel	Intronic	Intronic	Nonsense	Nonsense	Missense
Inheritance	Paternal	De novo	De novo	De novo	Unknown	Unknown	Unknown	Maternal (P9)	Unknown	Unknown
In silico prediction tools	Splice AI: Donor Loss 0.98 Donor Gain 0.91	Splice AI: Donor Loss 0.80	CADD: 31.0 Revel: 0.41 Alpha-Missense: 0.909	N/A	N/A	CADD: 23.4	N/A	CADD: 36	CADD: 36	Revel: 0.53 Alpha-Missense: 1
GnomAD v4.1 allele frequency	Absent	4/1441368 AF 0.0000028	Absent	Absent	Absent	2/1461100 AF 0.0000014	Absent	1/1596674 AF 6.263e^−7^	1/1596674 AF 6.263e^−7^	Absent
Pathogenicity criteria^c^	PM2 PVS1_strong = LP	PP3 PS2_sup = VUS	PM2 PS2_sup = VUS	PM2 PS2_sup PVS1_strong = LP	PM2 PM1 PM4_sup = VUS	PVS1_strong = VUS	PM2 PVS1_strong = LP	PM2 PVS1 = P	PM2 PVS1 = P	PM2 PM1 = VUS

*ADHD*, Attention deficit hyperactivity disorder; *AF*, allele frequency; *ASD*, autistic spectrum disorder; *ID*, intellectual disability; *LD*, learning difficulties; *LP*, likely pathogenic; *N/A*, not available; *NDD*, neurodevelopmental delay; *P*, pathogenic; *VUS*, variant of uncertain significance; *WM*, white matter.

a This individual was previously reported as P12.^29^

b *TAOK2* variants were annotated using the MANE Select NM_016151.4 transcript for TAOK2α isoform and NM_004783.4 transcript for TAOK2β isoform.

c Assessment based on the assumption of an established *TAOK2* gene-disease association.

## Data Availability

The authors confirm that the data supporting the findings of this study are available within the article and/or its supplemental data. Consent from individual patients precludes access to the raw genetic data.
