# Supplementary material for: Expanding the phenotype and genotype spectrum of TAOK1 neurodevelopmental disorder and delineating TAOK2 neurodevelopmental disorder
Source: Genet Med. Author manuscript; Available in PMC 2026 Aug 17. (PMC13479925; doi:10.1016/j.gim.2024.101348)
Supplement: Supplementary file 1 [file NIHMS2182537-supplement-supplemental_material.zip › 1-s2.0-S109836002400282X-mmc1.docx]

**Appendix: Lay Summary**

**Introduction: TAOK Proteins and Their Role in Developmental Disorders**

TAOK proteins are important for various functions in our body, such as communication between cells, organising cell structures, and brain development. Changes in the *TAOK1* gene, which alter the TAOK1 protein, are linked to a developmental disorder with unique facial features, low muscle tone, and feeding difficulties. Changes in the *TAOK2* gene, affecting the TAOK2 protein, are associated with autism and early obesity. Until now, there hasn’t been a clear description of a developmental disorder specifically related to *TAOK2* gene.

**Our Study**

We studied health and genetic information from individuals from several medical centres in different countries who have changes in the *TAOK1* or *TAOK2* genes. These changes were found using advanced genetic tests called exome and genome sequencing.

**Findings**

***TAOK1* Gene Changes**:

We studied 50 individuals with changes in the *TAOK1* gene (37 different *TAOK1* gene changes).

- **Common health problems**:
  - Developmental difficulties (such as delayed development, learning difficulties, autism, behavioural issues)
  - Larger head (83%)
  - Low muscle tone (58%)
- **Newly Reported health problems**:
  - Male genital abnormalities
  - Episodes of low blood sugar (hypoglycaemia)

**TAOK2 Gene Changes**:

We studied 10 individuals with changes in the *TAOK2* gene (nine different *TAOK2* gene changes).

- **Common health problems**:
  - Developmental difficulties (such as delayed development, learning difficulties, autism, behavioural issues)
  - Larger head (75%)
  - Autism (75%)
  - Overweight/obesity (70%)

**Key Discoveries**

- We report the largest group of individuals with *TAOK1*-related developmental disorder.
- We have identified 30 new changes in the *TAOK1* gene.
- We describe a new disorder related to *TAOK2* changes, characterised by developmental difficulties, autism, larger head, and obesity.
- **Similarities and Differences**:
  - Both *TAOK1* and *TAOK2* changes cause developmental difficulties.
  - Feeding difficulties and congenital abnormalities are more common with *TAOK1* changes.
  - Obesity is more common with *TAOK2* changes.
