# Supplementary material for: Expanding the phenotype and genotype spectrum of TAOK1 neurodevelopmental disorder and delineating TAOK2 neurodevelopmental disorder
Source: Genet Med. Author manuscript; Available in PMC 2026 Aug 17. (PMC13479925; doi:10.1016/j.gim.2024.101348)
Supplement: Supplementary file 1 [file NIHMS2182537-supplement-supplemental_material.zip › 1-s2.0-S109836002400282X-mmc4.pdf]

Supplementary data 1: Genetic testing methodology

NHS Whole genome sequencing (WGS) (*TAOK1* individuals P4, P5, P41 and P42)

WGS was performed by Illumina using the TruSeq PCRfree library preparation method followed by sequencing on the NovaSeq 6000 platform (Illumina Inc, San Diego, CA, USA). The variants were selected by the NHS Genomic Laboratory Hub following review of prioritised variants from the Genomics England interpretation (tiering) pipeline. It may include single nucleotide variants and small insertions/deletions in the virtual gene panel(s) classified as tier 1 or 2 and/or other types of prioritised variants that may be of relevance to the patient's phenotype. Tiered CNVs are high quality calls >2 kb derived from the proband only.

Deciphering Developmental Disorders (DDD) study (*TAOK1* individuals P11, P19, P20, P21, P25, P27, P28, P36, P39)

Samples were collected at the Wellcome Sanger Institute, and high-resolution exon-arrayCGH and trio exome sequencing was used to investigate the genetic causes of abnormal development. Likely diagnostic results were/are being reported to clinical teams, for validation (reportable variants are all confirmed using a secondary technique; Sanger, polymerase chain reaction or microarray).

100,000 Genomes Project (100kGP) (*TAOK1* individuals P12, P13, P14, P16, P17 and P18)

Trio whole genome sequencing by the 100kGP with analysis of Tier 1 and 2 variants of the panel(s) specified for each patient, and other variants as appropriate, followed by in-house Sanger sequencing confirmation where appropriate. Samples were sequenced using Illumina HiSeq 2500 instrument and data files were processed at the University of Cambridge High Performance Computing Service (HPC).

GEMINI (*TAOK1* individuals P1, P2 and P3)

Targeted gene sequencing test with 1722 genes associated with disorders that typically present in early life. Sequencing reads were mapped to the reference genome GRCh37 (hg19) and sorted for variant calling using Edico DRAGEN version 2.6.5 (Illumina). Opal clinical software identified relevant variants with a standard framework used to assess candidate variants for pathogenicity. A variant scientist, molecular geneticist, and genetic counselor reported all identified variants. Reporting to sites was similar to genomic sequencing. Reportable variants were all confirmed using a secondary technique (Sanger, polymerase chain reaction or microarray).

GeneDx (*TAOK1* individuals P22, P24, P24, P26, P35 and *TAOK2* individuals P2, P5, P7 and P10).

Using genomic DNA from the proband or proband and parent(s), the exonic regions and flanking splice junctions of the genome were captured using the IDT xGen Exome Research Panel v1.0 or v2.0 (Integrated DNA Technologies, Coralville, IA). Massively parallel (NextGen) sequencing was done on an Illumina system with 100bp or greater paired-end reads. Reads were aligned to human genome build GRCh37/UCSC hg19 and analyzed for sequence variants using a custom-developed analysis tool. Reported variants were confirmed, if necessary, by an appropriate orthogonal method in the proband and, if submitted, in selected relatives. Additional sequencing technology and variant interpretation protocol has been previously described[1]. The general assertion criteria for variant classification are publicly available on the GeneDx ClinVar submission page (http://www.ncbi.nlm.nih.gov/clinvar/submitters/26957/).

mRNA sequencing (*TAOK1* individual P50)

RNA is extracted from blood using a Qiagen PAXgene blood RNA kit. Reverse transcription is performed to convert the RNA into cDNA using a VILO SuperScript III RT-PCR system Life Technologies kit. Targeted PCR and Sanger sequencing is performed on the cDNA and the data is analysed using MutationSurveyor.

Supplementary references:

1- Retterer K, Juusola J, Cho MT, et al. Clinical application of whole-exome sequencing across clinical indications. Genet Med. 2016;18(7):696-704. doi:10.1038/gim.2015.148

Supplemental figure 1:

The *(TAOK1)* c.1909-7G>A intronic variant results in aberrant splicing

A. The reference *TAOK1* sequence with the c.1909-7 variant highlighted in a green frame.
B. Reference sequence for the *TAOK1* NM_020791.4 transcript (top) and electrophoretogram of mRNA sequencing for the *(TAOK1)* NM_020791.4:c.1909-7G>A variant (bottom) showing that the *(TAOK1)* NM_020791.4:c.1909-7G>A variant causes aberrant splicing with activation of a cryptic splice site resulting in intron inclusion of 5bp into exon 17, resulting in a frameshift and creating a termination codon 4 amino acids downstream p.(Glu637IlefsTer4). The transcript is predicted to undergo nonsense-mediated decay (NMD). Exon 16 in both the normal and abnormal transcripts is highlighted in a blue frame. Exon 17 in the normal transcript is highlighted in a yellow frame, and in the abnormal transcript is highlighted in a purple frame. The intron inclusion of 5bp is highlighted in A red frame.

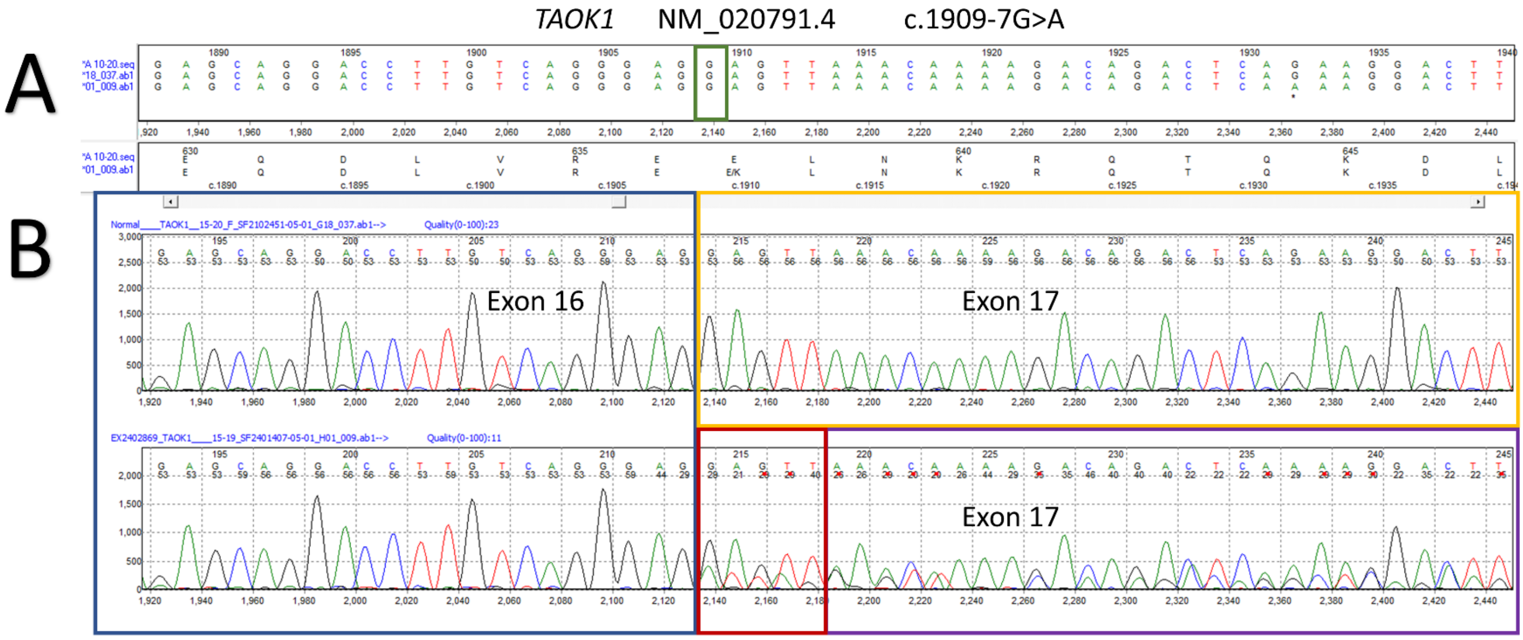

Supplemental figure 2:

Protein modelling with DDMut to assess the effect of variants on protein stability and dynamics. The TAOK1 and TAOK2 residues were modelled on the homologous TAOK2 PDB structure (PDB ID: 2GCD) in *Rattus norvegicus*, the closest structure for TAOK1 with an alignment score of 492 and an identity of 89.4%, due to lack of structural information on the human protein homologue.

A. *(TAOK1)* c.70C>A p.(Pro24Thr) with predicted destabilising effect: predicted Stability Change (ΔΔGStability) of -2.28 kcal/mol
B. *(TAOK1)* c.170A>C p.(Lys57Thr) with predicted destabilising effect: predicted Stability Change (ΔΔGStability) of -1.61 kcal/mol
C. *(TAOK1)* c.427C>T p.(His143Tyr) with predicted destabilising effect: predicted Stability Change (ΔΔGStability) of -0.36 kcal/mol
D. *(TAOK1)* c.499C>T p.(Leu167Phe) with predicted destabilising effect: predicted Stability Change (ΔΔGStability) of -1.1 kcal/mol
E. *(TAOK1)* c.512G>C p.(Gly171Ala) with predicted stabilising effect: predicted Stability Change (ΔΔGStability) of 0.24 kcal/mol
F. *(TAOK1)* c.554C>T p.(Thr185Met) with predicted destabilising effect: predicted Stability Change (ΔΔGStability) of -0.96 kcal/mol
G. *(TAOK1)* c.620A>T p.(Asp207Val) with predicted destabilising effect: predicted Stability Change (ΔΔGStability) of -0.08 kcal/mol
H. *(TAOK1)* c.656C>T p.(Ala219Val) with predicted stabilising effect: predicted Stability Change (ΔΔGStability) 0.9 kcal/mol
I. *(TAOK1)* c.750G>T p.(Trp250Cys) with predicted destabilising effect: predicted Stability Change (ΔΔGStability) of -0.86 kcal/mol
J. *(TAOK1)* c.785T>C p.(Leu262Pro) with predicted destabilising effect: predicted Stability Change (ΔΔGStability) of -3.03 kcal/mol
K. *(TAOK1)* c.878T>G p.(Leu293Arg) with predicted destabilising effect: predicted Stability Change (ΔΔGStability) of -1.76 kcal/mol
L. *(TAOK2)* NM_016151.4:c.463G>A p.(Gly155Arg) with predicted stabilising effect: predicted Stability Change (ΔΔGStability) of 0.22 kcal/mol

Colour legend: magenta: clash, Red: hydrogen bonds, yellow: ionic interactions, light green: aromatic contacts, dark green: hydrophobic, orange: polar contacts, sky blue: Van der Waals bonds. Stabilising mutations: (ΔΔG ≥ 0 kcal/mol). Destabilising mutations: (ΔΔG < 0 kcal/mol).

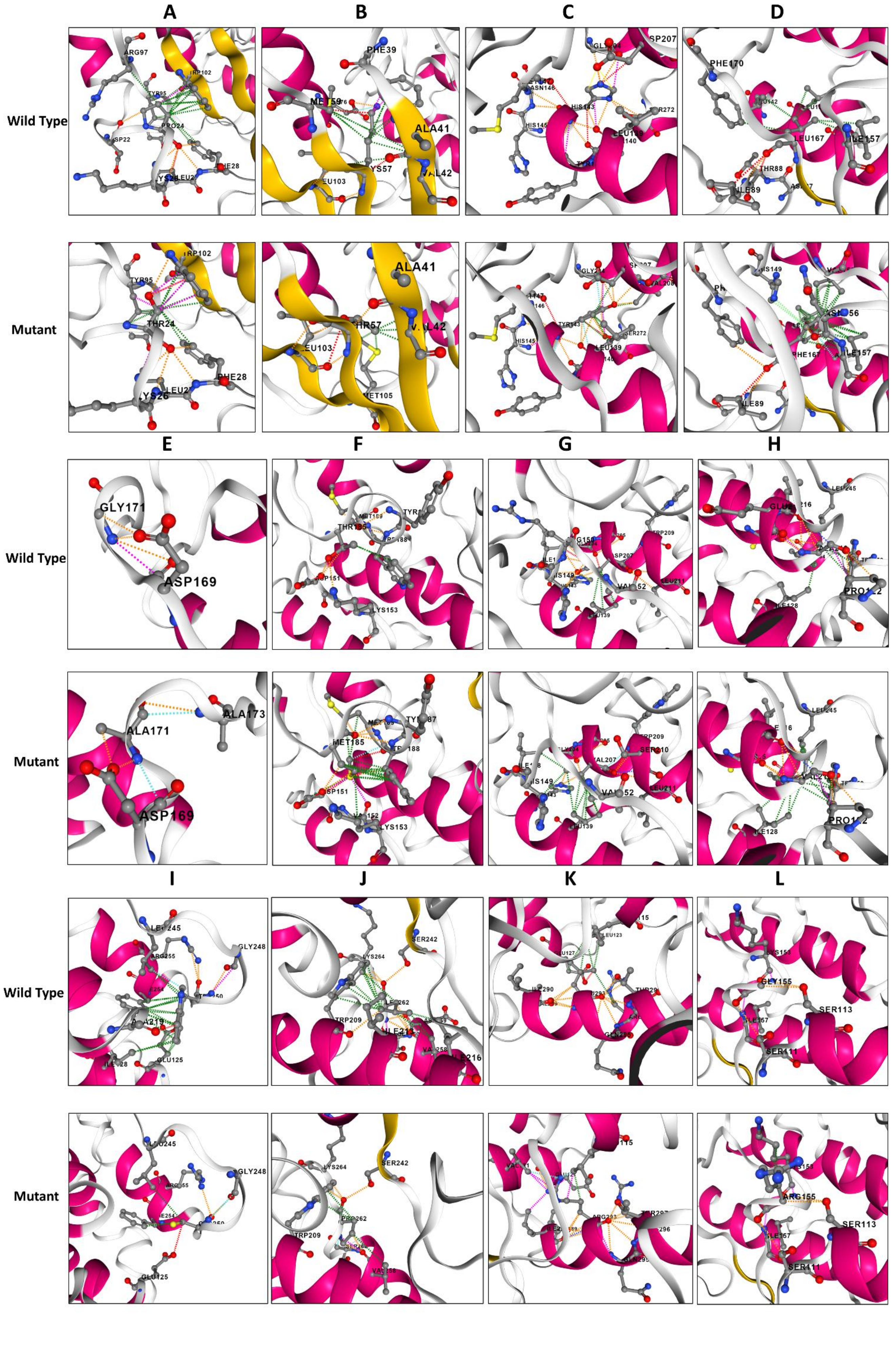
A
B
C
D
Wild Type
Mutant
E
F
G
H
Wild Type
Mutant
I
J
K
L
Wild Type
Mutant
